# Soil nitrous oxide measurement methods: Collar adaptors and comparison of manual versus automatic chamber headspace sample collection

**DOI:** 10.1002/jeq2.70224

**Published:** 2026-07-15

**Authors:** P. V. F. Machado, T. Vergara, K. Liu, J. Nimegeers, A. Glenn, F. Akhter, W. E. May

**Affiliations:** ^1^ Agriculture and Agri‐Food Canada Swift Current Saskatchewan Canada; ^2^ Agriculture and Agri‐Food Canada Brandon Manitoba Canada; ^3^ Agriculture and Agri‐Food Canada Indian Head Saskatchewan Canada

## Abstract

Manually operated non‐flow‐through non‐steady‐state chambers have been a dominant method for soil measurements of nitrous oxide (N_2_O) emission due to low cost, simplicity, and adaptability to different experimental designs. However, the use of automated dynamic non‐steady‐state chambers that deliver measurements in real‐time has increased in the literature. A systematic comparison of these two methods could assist with integration between the rich soil N_2_O knowledge established using manual chamber + gas chromatography (MC‐GC) versus the results increasingly generated by automatic chambers + trace gas analyzers (AC‐TGAs). The geometry of the system, a research‐specific requirement, can also affect the performance of chamber systems. Automatic chambers with site‐specific geometry could not be available or the cost could be prohibitive due to the advanced technology of such systems. The use of low‐cost geometry adaptors is an alternative, but their utilization must be preceded by performance validation. In this technical note, we compared daily N_2_O fluxes obtained with an AC‐TGA system versus MC‐GC. The comparison was also performed between two prototypes of rectangular adaptors (ADP‐1 and ADP‐2) and the standard round collar supplied with AC‐TGA. The regression approach returned a slope of 0.98 (confidence interval [CI] = 0.97–1.00) for AC‐TGA versus MC‐GC, indication of potential for generation of comparable datasets. The rectangular geometry adaptors demonstrated to be an alternative to meet research‐specific requirements in N_2_O studies. The prototype ADP‐1 resulted in equivalent fluxes to measurements performed with the standard round collar (slope = 1.00 [CI = 0.99–1.03]), but prototype ADP‐2 underestimated fluxes from 1% to 11%.

## Core Ideas

- Manual chamber + gas chromatograph (MC‐GC) was compared to automatic chamber + trace gas analyzer (AC‐TGA).
- Performance assessment was conducted for geometry adaptor prototypes to be used in automatic chamber systems.
- There is potential for acquisition of comparable datasets between MC‐GC and AC‐TGA.
- No chamber pressure and air mixing artifacts due to adaptors, but temperature was higher in some cases.
- Geometry adaptors were demonstrated to be an alternative to meet research‐specific requirements in N_2_O studies.

## Plain Language Summary

Manually operated greenhouse gas measurement chambers have been a dominant method for soil measurements of nitrous oxide (N_2_O). However, the use of automated dynamic chamber systems that deliver measurements in real‐time has increased in the literature. Thus, comparison between these two methods is needed for data integration. In automated chamber systems, the use of geometry adaptors might be needed to satisfy research‐specific requirements, but performance validation is needed. In this technical note, we compared daily N_2_O fluxes obtained with manually operated versus automated chambers. Rectangular geometry adaptor prototypes were also tested against a small round collar supplied with the automated chamber system. Our results indicated potential for generation of comparable N_2_O data between manual and automated chamber systems. In addition, geometry adaptors were a good alternative to meet the requirements of soil N_2_O research, but one of the prototypes underestimated N_2_O fluxes.

**Abbreviations**
AC‐TGA: automatic chamber + trace gas analyzer
ADP‐1: rectangular geometry adaptor 1
ADP‐2: rectangular geometry adaptor 2
CI: confidence interval
ECD: Electron Capture Detector
GC: gas chromatograph
GHG: greenhouse gas
MC‐GC: manual chamber + gas chromatography
OF‐CEAS: Optical Feedback–Cavity Enhanced Absorption Spectroscopy

## 1 INTRODUCTION

Agricultural systems that achieve high agronomic performance at reduced rates of emission of greenhouse gases (GHGs) are crucial to global food security and climate change mitigation. This can be seen in the increasing number of studies published in the literature on GHG emission in response to management of the agricultural system (e.g., Maaz et al., 2021; Machado, Farrell, Deen, et al., 2021; Reay et al., 2012). Among the GHGs, agriculture contributes to non‐carbon dioxide emissions, such as nitrous oxide, a powerful GHG (Drever et al., 2026; Frank et al., 2019). Nitrous oxide is mostly derived from soil biological reactions, such as nitrification and denitrification (Charles et al., 2017; Linton et al., 2020), and emissions are modulated by complex edaphic‐climatic factors and interactions (Glenn et al., 2021; Lemke et al., 2023).

Different techniques have been deployed for measurement of N_2_O under agricultural conditions, such as micrometeorological (e.g., Machado et al., 2020; Tariq et al., 2024) and chamber methods (e.g., Grace et al., 2020; Liu et al., 2024; Maier et al., 2022). A large number of publications from the literature are based on manually operated static chamber systems for determination of N_2_O fluxes, mostly due to the low cost, simplicity, and flexibility to adapt to different experimental designs (de Klein et al., 2020; Siddique et al., 2025). GHG sampling campaigns based on manual chambers are commonly composed of manual collection of headspace air from closed chambers at discrete deployment times, transfer of the samples to pre‐evacuated vials or bags, storage and shipping of samples to the laboratory, followed by analysis, commonly deploying gas chromatography (Harvey et al., 2020; Rapson & Dacres, 2014). Recently, the use of portable automated dynamic chamber systems for GHG measurements has increased in the literature (e.g., Hashemi et al., 2024; Kong et al., 2025). Since the bulk of our knowledge on soil N_2_O emissions from agricultural systems is based on manual chamber + gas chromatography (MC‐GC), research is needed to compare measurements obtained with this traditional method versus the automatic chamber + trace gas analyzer (AC‐TGA) solution. This could facilitate comparison between datasets collected with these two distinct chamber techniques.

Chamber design considerations, such as geometry, temperature, and pressure controls, are elements that need to be planned in detail for manual and automatic chamber systems, as they directly influence flux calculations (Clough et al., 2020; Rochette & Eriksen‐Hamel, 2008). The geometry of the measurement collars is a research‐specific feature of chamber systems. Research has shown that using circular collars for conditions where the N fertilizer is not evenly distributed but placed in a side‐ or middle‐row band can introduce uncertainty into the N_2_O flux determination (Clough et al., 2020; Rochette & Hutchinson, 2005). The alternative would be the deployment of flux corrections, strategic chamber placement, or the use of more collars per plot, to cover conditions with/without the N band (Cambareri et al., 2017; Charteris et al., 2020; Tenuta et al., 2023). Likewise, crop row spacing variation, which is dependent on agroecological characteristics, influences the concentration of N in the fertilizer band and consequently, the area that must be sampled by the chamber system. Hence, research‐specific design of chambers, which match the machinery used for seeding, N fertilizer application, and the overall management of the cropping system, could be an alternative when dealing with spatial variations in N_2_O fluxes (e.g., Olfs et al., 2018). For AC‐TGA, which commonly employs advanced portable analyzers connected to automated chambers, this can impose a challenge since collars of different geometry and sizes may not be available, or the cost associated with the purchasing or building of chambers of different shapes and sizes can be prohibitive due to the advanced technology deployed in such systems. The use of simple metal geometry adaptors in automatic systems has the potential to satisfy the site specificity required in soil N_2_O research at low costs. The objectives of this research were to (i) compare MC‐GC versus AC‐TGA measurements of daily N_2_O under field condition; and (ii) compare N_2_O emission measurements obtained using different rectangular collar adaptors in an AC‐TGA system. Our assessment also targeted relevant variables with potential to affect flux calculations, such as chamber temperature and pressure, and air mixing of the system.

## 2 MATERIALS AND METHODS

### 2.1 Site description and experimental design

This method comparison trial was conducted in a nonirrigated lawn area at the Swift Current Research and Development Centre (50°16′ N; 107°46′ W; elevation: 825 m). The site is located in the Mixed Grassland Ecoregion of Canada (TEC, 2021), in an area where Brown Chernozem soils are prevalent (SLC, 2010). The mean long‐term (1991–2020) annual precipitation for this site is 394 mm and the mean annual temperature is 4.1°C (ECCC, 2024), which are indicative of semiarid conditions. The experiment occurred in three phases: (i) from May 26 to July 18, 2025, comprising of 80 paired measurements taking place in four paired measurement collars (*n* = 8). This phase compared measurements with AC‐TGA, without the use of geometry adaptor, directly in a small 20‐cm diameter round collar supplied with the automatic system versus measurements with AC‐TGA using a small rectangular geometry adaptor (ADP‐1); (ii) from July 24 to August 20, 2025, comprising of 80 paired measurements in four paired measurement collars (*n* = 8). This phase compared direct measurements with AC‐TGA, without geometry adaptor, in a small 20‐cm diameter round collar versus measurements with AC‐TGA while using a large rectangular geometry adaptor (ADP‐2); and (iii) from May 26 to November 14, 2025, comprising of 285 paired measurements in four measurement collars. This phase compared MC‐GC versus AC‐TGA N_2_O measurements. Paired automated measurements and manual gas sample collection from the same gas chamber started concurrently. Detailed information on collar, adaptor, and measurement techniques can be found in Table 1.

**Core Ideas**

- Manual chamber + gas chromatograph (MC‐GC) was compared to automatic chamber + trace gas analyzer (AC‐TGA).
- Performance assessment was conducted for geometry adaptor prototypes to be used in automatic chamber systems.
- There is potential for acquisition of comparable datasets between MC‐GC and AC‐TGA.
- No chamber pressure and air mixing artifacts due to adaptors, but temperature was higher in some cases.
- Geometry adaptors were demonstrated to be an alternative to meet research‐specific requirements in N_2_O studies.

**TABLE 1:** Specifications of the different collars and adaptors tested in this research.

Treatment	Soil surface area (A) (cm^2^)	Total volume (V)^a^ (cm^3^)	Height (V/A) (cm)	Deployment time (Min)	Geometry (color^b^)	High frequency	Analytical technique^c^
MC‐GC	318 (*Ø* = 20 cm)	5895	18.5	10	Circular (reflective white)	No	Vials + GC
AC‐TGA	318 (*Ø* = 20 cm)	5895	18.5	3 & 10	Circular (reflective white)	Yes	Portable TGA
ADP‐1	644 (28.5 by 22.6 cm)	11384	17.7	3	Rectangular/circular top (reflective steel)	Yes	Portable TGA
ADP‐2	1226 (49.1 by 25.8 cm)	19117	15.6	3	Rectangular/circular top (reflective steel)	Yes	Portable TGA

Abbreviations: AC‐TGA, automatic chamber + trace gas analyzer; ADP‐1, rectangular geometry adaptor 1; ADP‐2, rectangular geometry adaptor 2; MC‐GC, manual chamber + gas chromatography.

^a^ System volume = automatic chamber + adaptor (when used) + 200 cm tubing + analyzer + collar offset + 1 cm^3^ septa adaptor system.

^b^ In AC‐TGA, MC‐GC, ADP‐1, and ADP‐2, a reflective white automatic dome‐shaped chamber was mounted either on a green 20‐cm diameter PVC collar (AC‐TGA and MC‐GC), or directly on the adaptors (ADP‐1 and ADP‐2).

^c^ GC = gas chromatograph equipped with an Electron Capture Detector (ECD); Portable TGA = OF‐CEAS N_2_O/H_2_O Trace Gas Analyzer.

### 2.2 Equipment and soil nitrous oxide measurements

**FIGURE 1:**
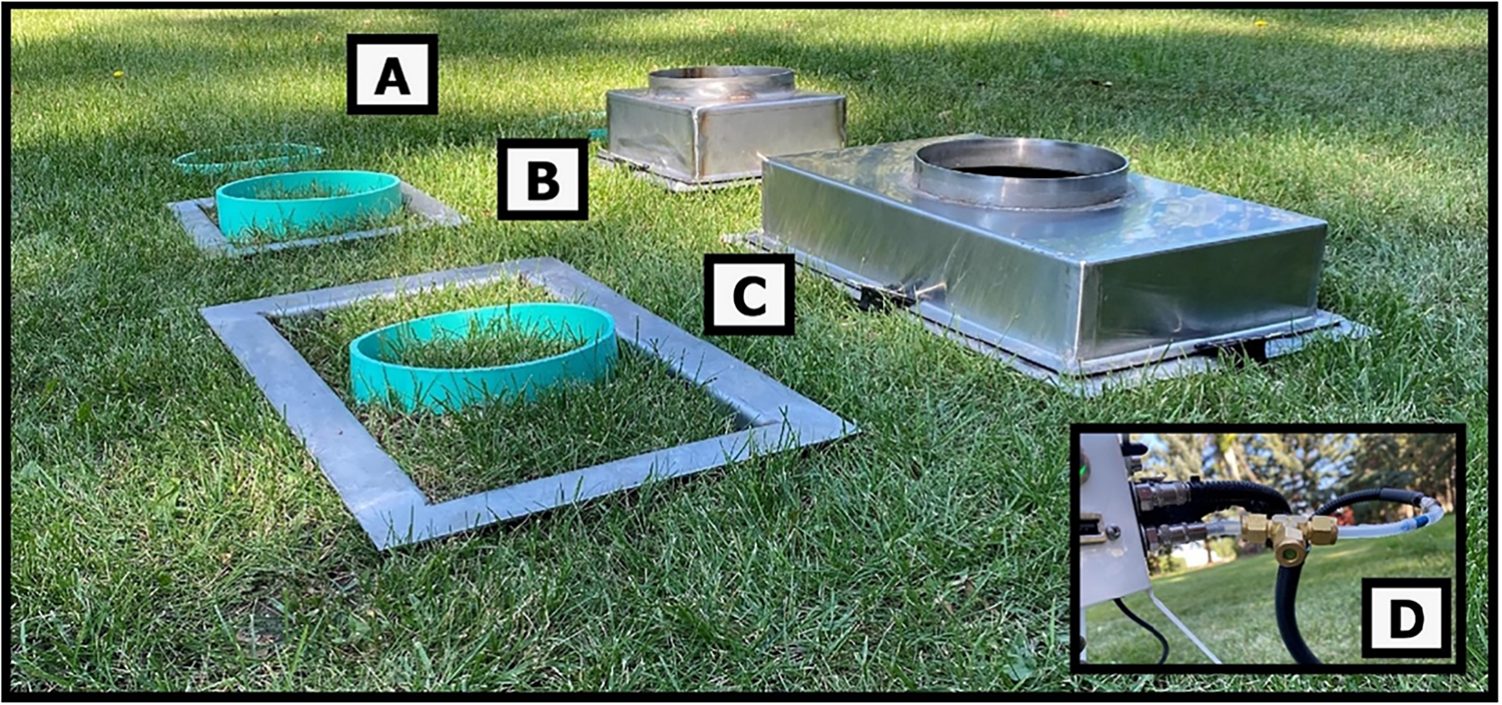
Round 20‐cm diameter collar used for the manual chamber + gas chromatography (MC‐GC) versus automatic chambers + trace gas analyzer (AC‐TGA) comparison (A), Adaptor 1 (B; collars on the left and metal adaptor on the right), and Adaptor 2 (C; collars on the left and metal adaptor on the right). The picture D shows the in‐line septa adaptor used for manual collection of soil greenhouse gas (GHG) samples concomitantly to the AC‐TGA measurement. ADP‐1 and ADP‐2 were constructed in reflective stainless steel, and sealing was enhanced with silicone sealant and water‐ and weather‐resistant foam rubber seal. Photo for visualization of the equipment only. The measurement site had four units of A, B, and C.

High N_2_O fluxes were stimulated with N fertilization and irrigation events to enable a wide range of emission for the test. Initially, four replicates of the 644 cm^2^ collar that is the corresponding base part for ADP‐1 were installed in the soil on May 25, 2025, with a 1 cm offset from soil surface to the top of the collar edge. After the installation, 5 g of urea‐N fertilizer was dissolved in 2 L of water and the volume was homogeneously applied per collar. In the following morning, 5 L of water was homogeneously applied in the 644 cm^2^ collars. After the irrigation implemented to stimulate high N_2_O fluxes, one 20‐cm diameter PVC collar was installed inside of each ADP‐1, with a 5 cm offset (Figure 1). Measurements started on May 26, 2025. For AC‐TGA, an automated chamber (model 8200–01S; LI‐COR Environmental) interfacing with a trace gas analyzer (model LI‐7820; LI‐COR Environmental) was used. The TGA used in this research employs Optical Feedback‐Cavity Enhanced Absorption Spectroscopy (OF‐CEAS) as the measurement technique. The automatic chamber was battery powered, vented, and connected in closed loop with the TGA. Measurements were initially performed as described for treatment AC‐TGA (Table 1) for 10 min deployment time and directly on the 20‐cm PVC collar. At the beginning of the measurement with AC‐TGA, a trace gas sampling kit (model 8100–664; LI‐COR Environmental) was installed between the automatic chamber and the OF‐CEAS analyzer to enable manual collection of gas samples concurrently with the automatic measurements. The manual samples were collected at 0, 5, and 10 min with syringes and needles, transferred to 12‐mL pre‐evacuated Exetainer vials, and stored until analysis. A gas chromatograph (GC) equipped with an Electron Capture Detector (ECD) was used for the analysis (model GC 8500; Scion Instruments Canada Limited, Edmonton, Canada). The samples were analyzed from July 14 to 18 and from November 28 to December 4, 2025. At the end of the 10 min measurements on the 20‐cm diameter PVC collar, the ADP‐1 was installed on the 644 cm^2^ collars, and automatic measurement was performed for 180 s to enable the ADP‐1 versus AC‐TGA comparison. For the ADP‐1 versus AC‐TGA comparison, five paired data were discarded from the regression analysis due to unforeseen wildlife excreta deposition in one of the measurement collars. The affected collar was repositioned with fertilizer and water management performed as described above, and measurements were resumed afterward.

The second stage of the trial was similar, but only the comparison between AC‐TGA versus ADP‐2 was performed (no manual sampling). For the larger adaptor, methods were similar to ADP‐1, but higher urea‐N rate (10 g dissolved in 2 L of water) and larger irrigation volume were used (8 L of water the next morning after N fertilization). Finally, additional measurements were conducted to increase the number of paired data for the comparison between MC‐GC and AC‐TGA between August 20 and November 14, 2025, with fertilization and irrigation as described above for the AC‐TGA versus MC‐GC comparison. In all phases, the sampling campaigns were conducted in the mid‐morning (from 9:00 a.m. to 12:00 p.m.) and mid‐afternoon (from 1:00 p.m. to 4:00 p.m.), at approximately equal proportions, to enable a wide range of climatological conditions for our test. In Phases i and iii, N fertilizer application and irrigation were repeated throughout the experimental period, with collar replacement into a new undisturbed area. This strategy enabled a wide range of N_2_O fluxes for the test. Specifically, this process occurred on June 17 and 26 for Phase i, and September 7 (in half of the collars), September 15 (in half of the collars), and October 7 and 17 for Phase iii. No reapplication of fertilizer occurred for Phase ii. Graphical visualization of ADP‐1, ADP‐2, and the manual sampling line adaptor is presented in Figure 1.

### 2.3 Flux calculations and ancillary measurements

Flux calculations for the high‐frequency automatically collected data were performed using SoilFluxPro 5 Software (LI‐COR Environmental). The best fit was selected based on the coefficient of determination, and maximized deadband determination was performed, for the whole run, using the Guidance application. Chamber temperature measurements, used in both manual and automatic calculations, were performed with the built‐in thermistor from the automatic chamber. System pressure was measured with the built‐in sensor on the OF‐CEAS TGA. The calculation of fluxes from the manual measurements was performed as described in Venterea et al. (2020). To avoid introducing bias into the AC‐TGA versus MC‐GC comparison, flux calculations for both treatments were exclusively based on linear models.

### 2.4 Data and statistical analysis

The evaluation of the relationship between AC‐TGA versus MC‐GC, AC‐TGA versus ADP‐1, and AC‐TGA versus ADP‐2 was conducted with a regression analysis. The distribution of the residuals of the regressions, accessed with a quartile–quartile (*Q–Q*) plot, demonstrated an overall heavy tail shape/distribution for all the comparisons, an indication of more outliers/extreme values than a normal distribution and/or high concentration of values near the mean. The data, for all paired comparisons, exhibited heteroscedasticity according to the Breusch–Pagan test (*p* < 0.05). Thus, the nonparametric Passing–Bablok Method Comparison Regression using the *mcr* package in R was used (Potapov et al., 2024). Each individual daily flux obtained with AC‐TGA was plotted against MC‐GC, ADP‐1, and ADP‐2. The slope was evaluated for its difference from 1. For AC‐TGA versus MC‐GC, which had 285 pairs of data, the regression presented low data support for fluxes > 300 g N_2_O‐N ha^−1^ day^−1^, with only five paired observations; and for fluxes ranging from 100 to 300 g N_2_O‐N ha^−1^ day^−1^, with 33 observations. The majority, or 87% of the data (*n* = 247 paired points), presented good coverage in a range of fluxes from 0 to 100 g N_2_O‐N ha^−1^ day^−1^. Thus, two regressions were conducted for AC‐TGA versus MC‐GC: (i) all the 285 pairs of data; and (ii) 247 pairs of data representing the range of the regression with good coverage. For evaluation of chamber pressure in response to the use of adaptors, the difference between chamber pressure prior to deployment (T0, at 0 s; chamber open) and at the end of the deployment (T180, at 180 s; chamber closed) was calculated. Similarly, the difference between chamber temperature at T0 and T180 was calculated. This approach assumed that significant changes in chamber pressure and temperature in such a short deployment time (i.e., 180 s) could indicate a system that is prone to introduction of bias into the N_2_O flux calculations due to chamber pressure and temperature artifacts. The Wilcoxon signed‐rank test using the *coin* package in R (Hothorn et al., 2006), with the Pratt method when there were ties, was used to identify differences in chamber pressure and temperature before and at the end of the deployment time. The same test was used to identify differences between ADP‐1 versus AC‐TGA, ADP‐2 versus AC‐TGA, and AC‐TGA versus MC‐GC. The MGG (Miao–Gel–Gastwirth) test using the *lawstat* package in R tested symmetry in distribution of the data (Hui et al., 2008) prior to running the Wilcoxon signed‐rank test. Evaluation of the model fitting for slope calculations of the change in N_2_O concentration per time (dC/dT), by using the coefficient of determination, was performed for assessment of air mixing effects in response to use of ADP‐1 and ADP‐2. This assumed that poor air mixing would naturally create heterogeneity in N_2_O concentration increases during the measurement deployment, which would negatively affect fitting performance when calculating dC/dT and the calculated N_2_O fluxes.

## 3 RESULTS AND DISCUSSION

### 3.1 Manual versus automatic measurements

**FIGURE 2:**
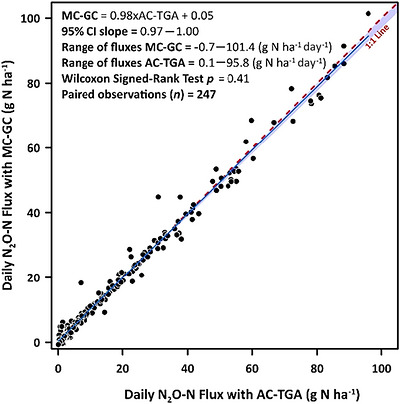
Regression analysis of the daily N_2_O fluxes < 100 g N_2_O‐N ha^−1^ for the automatic chamber connected to a trace gas analyzer system (AC‐TGA) versus the manual chamber, with samples analyzed in a gas chromatograph (MC‐GC). The blue shaded area around the regression line represents the 95% slope confidence interval (CI). High data density occurred for the range of fluxes < 100 g N_2_O‐N ha^−1^.

The daily N_2_O fluxes from the 285 paired AC‐TGA versus MC‐GC comparisons ranged from −0.7 to 513.1 g N_2_O‐N ha^−1^. These data coverage are within or surpassing common values reported in the literature for different agricultural systems, under a range of climatological conditions (e.g., An et al., 2021; Lemke et al., 2023; Pelster et al., 2021; Tenuta et al., 2023). The regression analysis to only consider the high data density range of the regression (i.e., 0–100 g N_2_O‐N ha^−1^ day^−1^), presented a slope of 0.98, with a confidence interval (CI) ranging from 0.97 to 1.00 (Figure 2). In addition, the Wilcoxon signed‐rank test to compare between AC‐TGA and MC‐GC was not significant for fluxes ranging from 0 to 100 g N_2_O‐N ha^−1^ day^−1^ (*p* = 0.41; Figure 2). The regression analysis with all the data, including in the ranges of the regression that presented low data density (i.e., >100 g N_2_O‐N ha^−1^ day^−1^), presented a 95% CI ranging from 0.96 to 0.98 (Figure S1), a result potentially influenced by high leverage and the influence of outliers in ranges of the regression with insufficient data coverage. Additional research to evaluate agreement between AC‐TGA versus MC‐GC under high fluxes > 100 g N_2_O‐N ha^−1^ day^−1^ are still needed and should also investigate the calibration performance of the gas chromatograph and trace gas analyzer under this specific range of high fluxes.

Overall, our results are evidence that determination of N_2_O fluxes with portable, automatic chamber systems could inherently result in values that are comparable to manual measurements, at a wide range of N_2_O fluxes. Notably, by sampling concomitantly to AC‐TGA with a septa adaptor (Figure 1D), our approach equalized some of the major sources of error with potential to confound the comparison. Spatial variation in N_2_O emission and chamber design considerations, large sources of variation in N_2_O studies (Ashiq et al., 2021; Machado, Farrell, Wagner‐Riddle, et al., 2021) were, for example, kept the same between the two methods. Besides, by sampling at the same time for manual and the automatic measurements, diurnal variation errors (e.g., Alves et al., 2012; Reeves & Wang, 2015) were also normalized between MC‐GC and AC‐TGA. The comparison was thus influenced by other aspects such as sample collection and handling (syringe/needles vs. real‐time measurements), storage (with storage vs. real‐time), analytical method (GC analysis vs. OF‐CEAS), and flux calculation (high‐frequency vs. discrete measurements).

The study by Mendis et al. (2026) also evaluated agreement between OF‐CEAS and manual chamber measurements under field conditions, using two distinct chamber systems (a rubber lid for manual GC and an automatic chamber for OF‐CEAS), with measurements conducted no more than 15 min apart. In contrast, our approach minimized potential confounding factors by using gas samples originating from the same automatic chamber system for both AC‐TGA and MC‐GC, with concomitant measurements. Despite methodological differences, our findings are consistent with and complement the findings reported by Mendis et al. (2026).

Similarities as to how the analytical systems deal with water vapor in the sample might have influenced our results. The portable OF‐CEAS analyzer used in our research performs a water vapor correction, consequently reporting the N_2_O as a mole fraction in dry air (Kong et al., 2025). Similarly, the GC used in this research for analysis of manually collected samples is equipped with two channels, the first one with two detectors (thermal conductivity detector and flame ionization detector, for CO_2_ and CH_4_ determination, respectively) and the second one with an ECD for N_2_O measurements. The N_2_O detection happens after separation of the gas sample from water vapor, which is backflushed to a vent. In summary, the comparison between treatments AC‐TGA and MC‐GC is evidence that integration of N_2_O data generated with the two distinct methods could be possible, particularly when the manual chamber system is designed according to modern guidelines that ensure proper sealing, air mixing, chamber temperature stability, and lack of pressure oscillations in the system. Additional research is still needed to compare manual chamber measurements against other analytical methods with potential to be used in portable analyzers deployed in automatic soil N_2_O chamber systems (e.g., Niu et al., 2025; Stiefvater et al., 2023).

### 3.2 Geometry adaptors: an alternative to achieve the site‐specific requirements of soil nitrous oxide studies

The daily N_2_O fluxes for the rectangular adaptor prototypes trials ranged from 0.5 to 454.3 and 0.6 to 265.4 g N_2_O‐N ha^−1^ day^−1^ for ADP‐1 and ADP‐2, respectively. Similar to what was reported in the previous subsection, this range of daily N_2_O fluxes, stimulated with high N rates and irrigation events in our research, is within or surpassing common reported values for a range of agricultural systems in semiarid climates (e.g., An et al., 2021; Dungan et al., 2023; Lemke et al., 2023), subhumid ecozones (Tenuta et al., 2023; Wood et al., 2024), and more mesic environments (e.g., Chatterjee et al., 2025; Pelster et al., 2021).

Chamber pressure artifacts were not detected in ADP‐1 or ADP‐2, and the pressure between pre‐deployment (with the chamber open) and at the end of the measurement (with the chamber closed) was not different than zero in any of the tested prototypes (*p* > 0.05; Figure S2). Similarly, the two datasets collected without adaptors also had nonsignificant differences in pressure between T0 and T180 measurements (*p* > 0.05; Figure S2). Chamber temperature controls were demonstrated to be superior when the reflective white, dome‐shaped automatic chamber was mounted directly into the round 20 cm collar (i.e., without adaptor). This treatment did not result in temperature increases between T0 and T180 for mid‐morning and mid‐afternoon collected data (Figure S3). The ADP‐1 and ADP‐2 also did not result in temperature increases for the mid‐morning measurements (*p* > 0.05; Figure S3) but were on average 2.3°C and 1.8°C warmer than T0 at 180 s for mid‐afternoon campaigns, respectively (*p* < 0.05; Figure S4).

Our data analysis did not provide evidence of poor air mixing when using the rectangular adaptors. We assumed that any potential artifact related to poor air mixing could result in temporal instability in N_2_O concentration increases after chamber closure and errors in flux determination. Thus, the fitting performance for dC/dT calculations was used in combination to N_2_O flux comparisons between AC‐TGA versus ADP‐1 and AC‐TGA versus ADP‐2 for the assessment. This assumed that the automatic chamber mounted directly on a 20‐cm diameter circular collar (AC‐TGA) would not be prone to poor air mixing artifacts since the chamber air, actively pumped to and from the N_2_O analyzer, was continuously circulated in a small volume in a dome‐shaped closed system (Table 1). Comparatively with AC‐TGA, a variation in N_2_O concentration that does not follow a similar trend in N_2_O concentration after chamber closure could indicate air mixing imperfections. That effect would naturally influence the fitting performance when calculating dC/dT for flux determinations and the final calculated flux.

In our study, 92% and 93% of the dC/dT calculations presented a coefficient of determination >0.90 for AC‐TGA and ADP‐1, respectively (Table 2). The N_2_O concentration also increased similarly to AC‐TGA when using ADP‐2, with 94% of the dC/dT slope presenting a coefficient of determination superior to 0.90 (with 70% presenting an *R* ^2^ > 0.99; Table 2). Our study only investigated adaptors that would increase the total volume of the system from 1.9 to 3.2 times in comparison to measurements performed directly on the small circular collar (Table 1). Future research on air mixing performance of larger adaptors could require a more detailed assessment, for example, with the installation of multiple sampling ports in the system to enable determination of an N_2_O concentration gradient in the chamber and adaptor profile, particularly targeting near the soil surface and locations potentially subjected to poor air mixing due to the geometry of the adaptor.

**TABLE 2:** Regression fit performance and ancillary measurements from treatments.

Regression fit							
Method	*R* ^2^ (dC/dT fit)			Method	*R* ^2^ (dC/dT fit)		
	>0.90 (>0.99)	0.70–0.90	<0.70		>0.90 (>0.99)	0.70–0.90	<0.70
**AC‐TGA**	92% (67%)	8%	0%	**AC‐TGA**	94% (73%)	5%	1%
**ADP‐1**	93% (64%)	4%	3%	**ADP‐2**	93% (70%)	7%	0%

Ancillary measurements^a^									
Method	Chamber P (range)	∆P (range)	Chamber T (range)	∆T (range)	Method	Chamber P (range)	∆P (range)	Chamber T (range)	∆T (range)
**AC‐TGA**	92.02 to 93.41	−0.03 to 0.04	14.5 to 34.5	−1.5 to 1.5	**AC‐TGA**	92.09 to 93.99	−0.08 to 0.03	12.6 to 33.6	−1.9 to 0.9
**ADP‐1**	92.02 to 93.39	−0.02 to 0.03	14.1 to 33.3	−1.3 to 4.8	**ADP‐2**	92.04 to 93.98	−0.04 to 0.05	12.3 to 35.1	−0.8 to 3.6

*Note*: AC‐TGA = automatic chamber + trace gas analyzer system using a 20‐cm diameter PVC collar; ADP‐1 = AC‐TGA with a 644 cm^2^ rectangular geometry adaptor; and ADP‐2 = AC‐TGA with a 1226 cm^2^ rectangular geometry adaptor.

^a^ P = Pressure (kPa); T = Temperature (°C); ∆P = chamber pressure difference between T = 180 s (end of deployment; chamber closed) and T = 0 s (pre‐deployment; chamber open) in kPa; ∆T = chamber temperature difference between T = 180 s (end of deployment; chamber closed) and T = 0 s (pre‐deployment; chamber open); detailed chamber pressure and temperature analysis is presented in Figure S2, S3, and S4.

**FIGURE 3:**
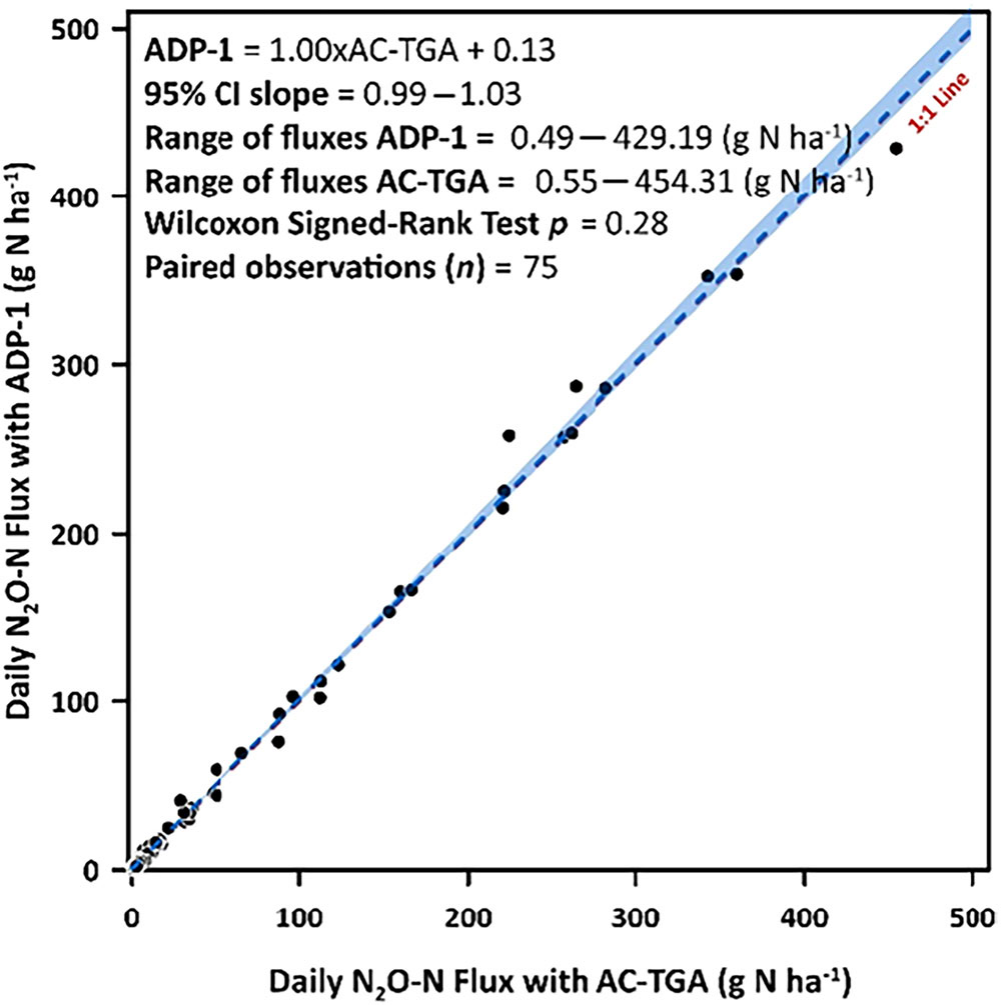
Regression analysis of the daily N_2_O fluxes for the automatic chamber connected to a trace gas analyzer system (AC‐TGA) versus the automatic chamber and trace gas analyzer using the prototype adaptor 1 (ADP‐1). The blue shaded area around the regression line represents the 95% slope confidence interval (CI).

Regarding the daily N_2_O fluxes, the overall performance was equivalent between ADP‐1 and AC‐TGA (Figure 3), irrespective of the 2.3°C warmer chamber temperature on average for ADP‐1 for measurements performed in the mid‐afternoon. That was likely because (i) the difference in temperature between T0 and T180s never surpassed 5°C h^−1^, a threshold where temperature artifacts on N_2_O flux calculation become more pronounced (Clough et al., 2020; Parkin & Venterea, 2010); and (ii) high‐frequency chamber temperature was measured and used in the N_2_O flux calculation. The regression analysis returned a slope of 1.00, with a CI ranging from 0.99 to 1.03 and, as such, not different than 1. In addition, the Wilcoxon signed‐rank test was not significant for ADP‐1 versus AC‐TGA (p = 0.28; Figure 3). The two statistical approaches used in the data evaluation of AC‐TGA versus ADP‐1 confirmed that using such a prototype to adapt geometry would not introduce bias into the daily N_2_O emission determination.

**FIGURE 4:**
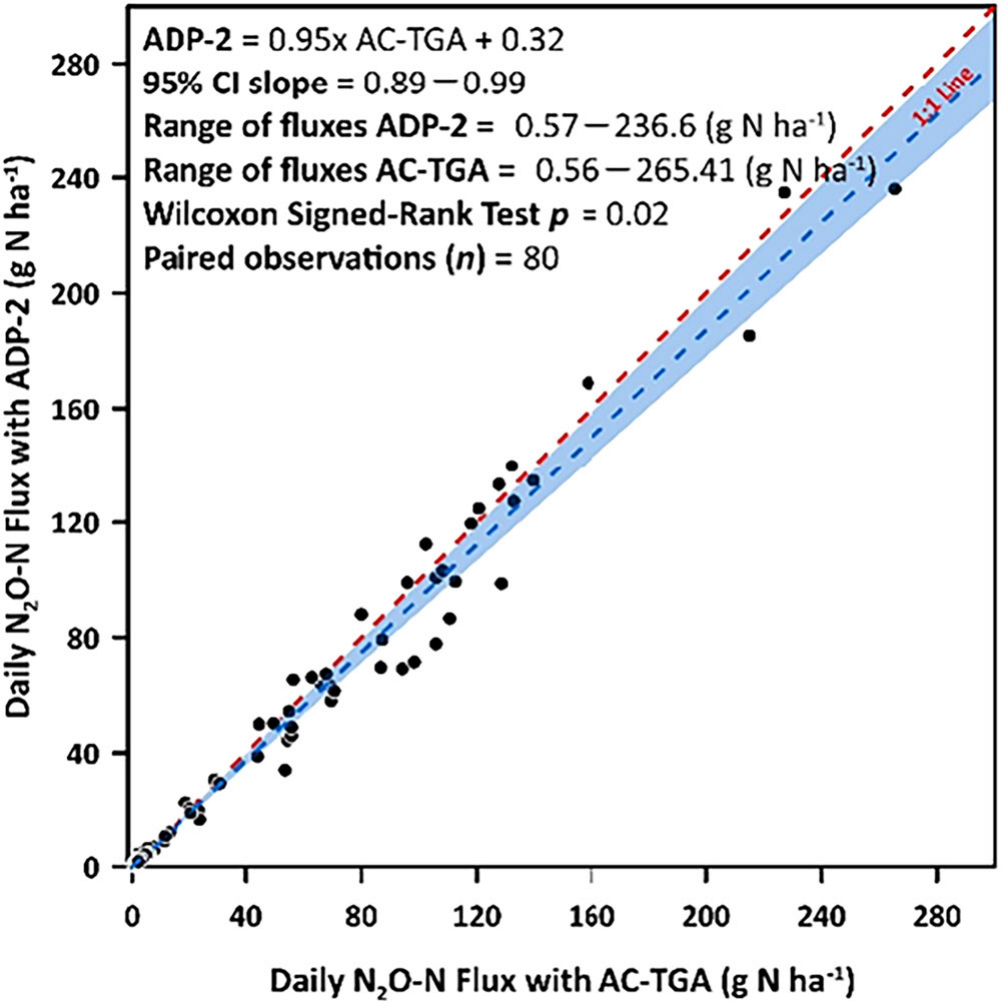
Regression analysis of the daily N_2_O fluxes for the automatic chamber connected to a trace gas analyzer system (AC‐TGA) versus the automatic chamber and trace gas analyzer using the prototype adaptor 2 (ADP‐2). The blue shaded area around the regression line represents the 95% slope confidence interval (CI).

The results were different for the comparison between ADP‐2 versus AC‐TGA (Figure 4). The two statistical methods used in the comparison provided evidence that ADP‐2 was prone to underestimation of fluxes, whereas the Wilcoxon signed‐rank test indicated significant difference between ADP‐2 versus AC‐TGA (*p*‐value = 0.02), the slope of the regression was 0.95, with a tendency for underestimation of fluxes when using ADP‐2 ranging from −1% to −11% (Figure 4). The wider range of CI for the slope was expected since ADP‐2, although in the same location as AC‐TGA, had an area 3.9 times larger than the 20‐cm diameter collar, and spatial variation in N_2_O emissions can occur even within short distances in the landscape (Ball et al., 2000; McDaniel et al., 2017). However, the fact that the slope was significantly different than 1 and the statistical test indication of a significant difference between ADP‐2 and AC‐TGA could be evidence of imperfections related to sealing in ADP‐2. We speculate that further structural enhancement, particularly targeting the base of ADP‐2 to improve rigidity, and reduce bending and gaps, is needed to ensure an airtight seal. The second, enhanced version of the prototype ADP‐2 will require additional performance assessment.

When placed in perspective, the use of geometry adaptors to satisfy the site‐specific requirements of N_2_O research resulted in a range of uncertainty that was smaller than other potential sources of measurement error in N_2_O research such as spatial variation (Hénault et al., 2012; Machado, Farrell, Wagner‐Riddle, et al., 2021), frequency of sampling (Barton et al., 2015; Van der Weerden et al., 2013), diurnal variation errors (Alves et al., 2012; Machado et al., 2019), and chamber design artifacts (Bain et al., 2005; Rochette & Eriksen‐Hamel, 2008). To reduce spatial variation in N_2_O emissions, an aspect with potential to confound our method comparison, our research was conducted in a homogeneous lawn area. Additional research is needed to compare cumulative N_2_O emissions between methods, as our study focused on daily fluxes, and to evaluate how geometry adaptors influence spatial variation in N_2_O emissions across a range of crop spacing widths, N concentrations, and edaphic‐climatic conditions.

## 4 CONCLUSIONS

This methods approach enabled comparisons between an OF‐CEAS‐based automatic real‐time chamber system versus manual measurements analyzed with an ECD, eliminating some of the measurement errors that could have confounded the comparison. As the scientific basis for flux calculations is inherently similar, our results provided evidence that comparable results can be generated using both methods. Comparability would depend on features such as chamber design considerations, analytical method, maintenance and calibration of the automatic, portable system, frequency of measurement, diurnal variation, and statistical and flux calculation considerations. Our results demonstrated that use of low‐cost geometry adaptors is a viable alternative to satisfy research‐specific needs in N_2_O research, but testing is recommended to ensure performance. Overall, cost‐effective adaptation of automated systems to site‐ and experiment‐specific scenarios can maintain data comparability with traditional manual chamber measurements.

## AUTHOR CONTRIBUTIONS

**P. V. F. Machado**: Conceptualization; data curation; formal analysis; funding acquisition; investigation; methodology; project administration; resources; supervision; validation; visualization; writing—original draft; writing—review and editing. **T. Vergara**: Investigation; methodology; writing—review and editing. **K. Liu**: Conceptualization; funding acquisition; methodology; writing—review and editing. **J. Nimegeers**: Supervision. **A. Glenn**: Conceptualization; funding acquisition; investigation; methodology; writing—review and editing. **F. Akhter**: Methodology; writing—review and editing. **W. E. May**: Methodology; writing—review and editing.

## CONFLICT OF INTEREST STATEMENT

The authors declare no conflicts of interest.

## Supporting information



Supplementary Material
