# Supplementary material for: Soil nitrous oxide measurement methods: Collar adaptors and comparison of manual versus automatic chamber headspace sample collection
Source: J Environ Qual. 2026 Jul 15;55(4):e70224. doi: 10.1002/jeq2.70224 (PMC13373487; doi:10.1002/jeq2.70224)
Supplement: Supplementary file 1 — Supplementary Material [file JEQ2-55-0-s001.docx]

**Supplementary File**


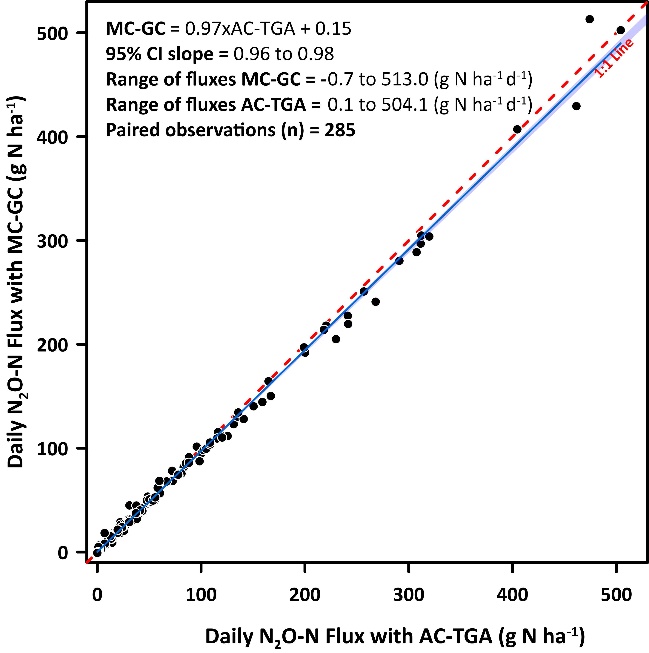


Figure S1. Regression analysis of the daily N_2_O fluxes for the automatic chamber connected to a trace gas analyzer system (AC-TGA) versus the manual chamber, with samples analysed in a gas chromatograph (MC-GC). The blue shaded area around the regression line represents the 95% slope confidence interval (CI).


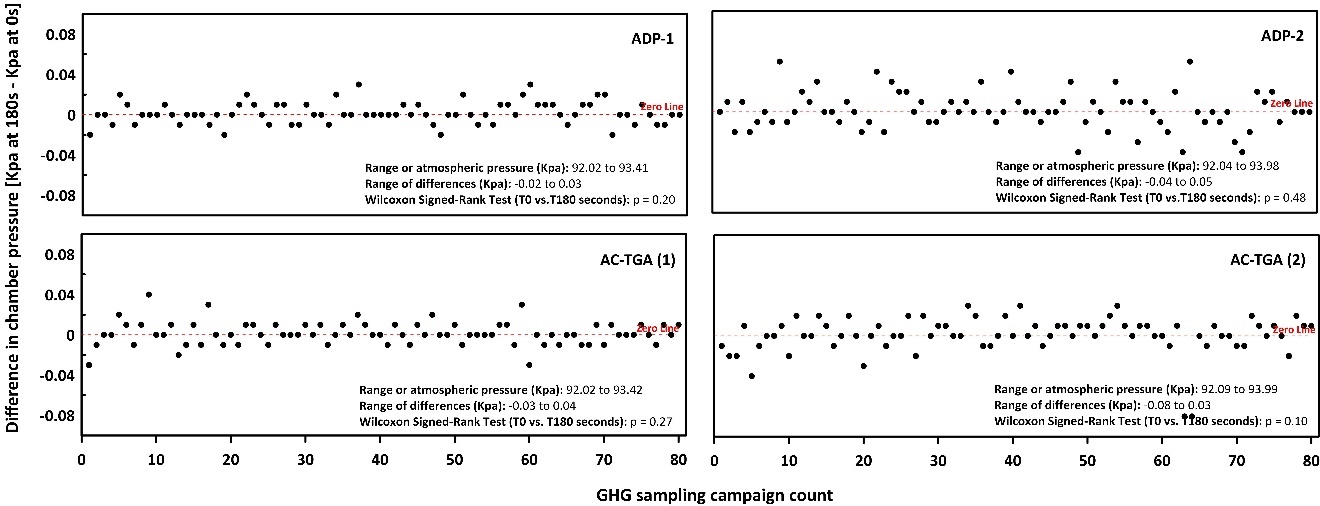


Figure S2. Difference in pressure between the beginning of deployment (with chamber open) and the end of the deployment at 180s (with the chamber closed). ADP-1 and ADP-2 are the two rectangular geometry adaptor prototypes tested in this study. Measurements were always paired with ADP-1/ADP-2 versus the automatic chamber + trace gas analyzer without adaptor (AC-TGA) (i.e., 2 datasets were collected with AC-TGA). The difference between T0 and T180 was symmetric for all datasets.


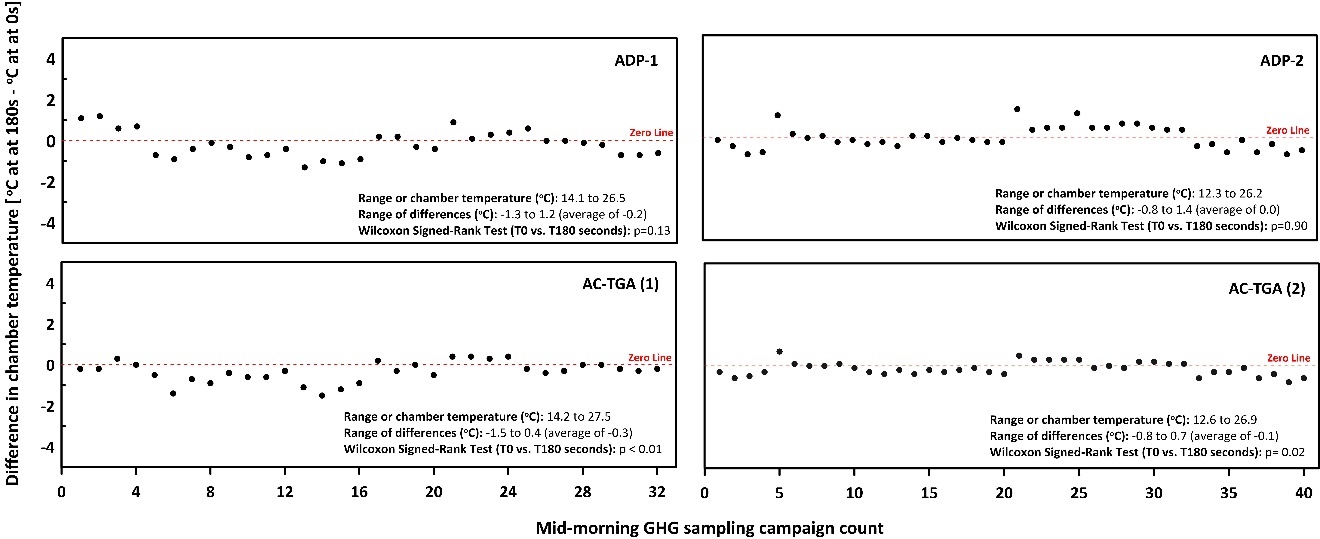


Figure S3. Difference in temperature between the beginning of deployment (with chamber open) and the end of the deployment at 180 seconds (with the chamber closed) for mid-morning sampling campaigns. ADP-1 and ADP-2 are the two rectangular geometry adaptor prototypes tested in this study. Measurements were always paired with ADP-1/ADP-2 versus the automatic chamber + trace gas analyzer without adaptor (AC-TGA) (i.e., 2 datasets were collected with AC-TGA). The difference between T0 and T180 was symmetric for all datasets.


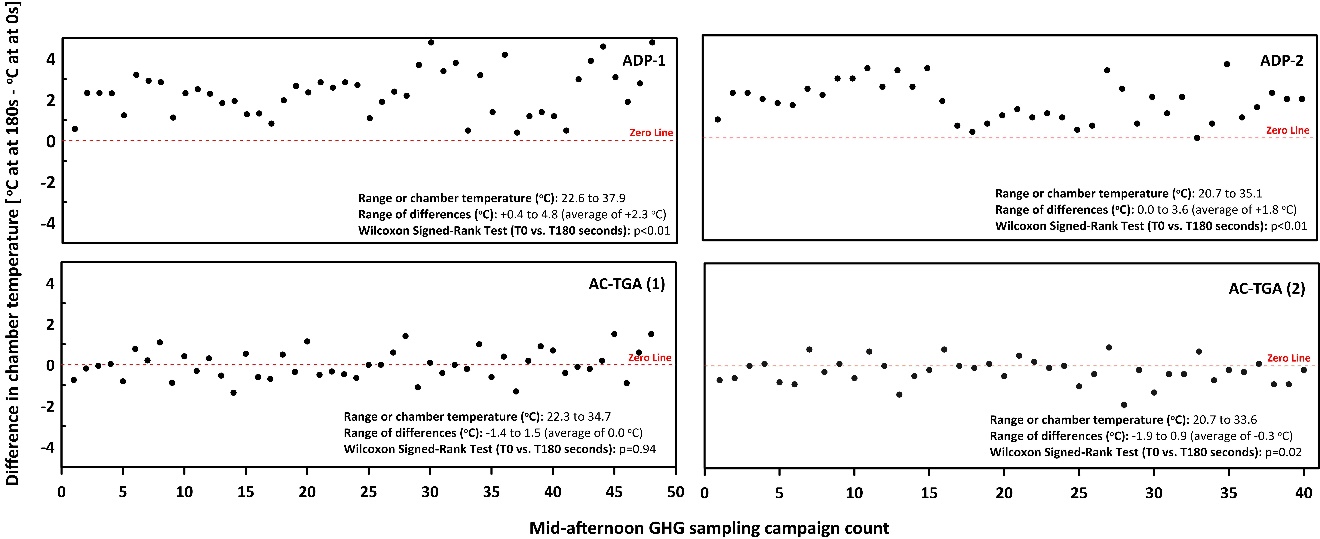


Figure S4. Difference in temperature between the beginning of deployment (with chamber open) and the end of the deployment at 180 seconds (with the chamber closed) for mid-afternoon sampling campaigns. ADP-1 and ADP-2 are the two rectangular geometry adaptor prototypes tested in this study. Measurements were always paired with ADP-1/ADP-2 versus the automatic chamber + trace gas analyzer without adaptor (AC-TGA) (i.e., 2 datasets were collected with AC-TGA). The difference between T0 and T180 was symmetric for all datasets.
